# Effectiveness of provider communication training for increasing human papillomavirus vaccine initiation at a safety-net health system

**DOI:** 10.1016/j.pmedr.2024.102660

**Published:** 2024-02-19

**Authors:** Rachel J. Meadows, Aaron W. Gehr, Yan Lu, Grace Maynard, Idara N. Akpan, Tanjila Taskin, Kimberly G. Fulda, Divya Patel, Sarah Matches, Rohit P. Ojha, Erika L. Thompson

**Affiliations:** a Center for Epidemiology & Healthcare Delivery Research, JPS Health Network, Fort Worth, TX, USA; b Department of Population & Community Health, School of Public Health, University of North Texas Health Science Center, Fort Worth, TX, USA; c North Texas Primary Care Practice-Based Research Network (NorTex), Department of Family Medicine and Osteopathic Manipulative Medicine, Texas College of Osteopathic Medicine, University of North Texas Health Science Center, Fort Worth, TX, USA; d Department of Epidemiology, Human Genetics and Environmental Science, UTHealth Houston School of Public Health in Austin, Austin, TX, USA; e Department of Pediatrics & Women’s Health, Texas College of Osteopathic Medicine, University of North Texas Health Science Center, Fort Worth, TX, USA

**Keywords:** Papillomavirus vaccines, Papillomavirus Infections/prevention & control, Vaccination, Provider communication, Primary prevention

## Abstract

**Background:**

Strong provider recommendation can increase uptake of human papillomavirus (HPV) vaccination. Therefore, we developed and implemented a provider education intervention on communication strategies for recommending HPV vaccination with clinic-level audit and feedback (HPV: Communicating about HPV to Adults and Teens [HPV CHAT]). We aimed to evaluate the effect of HPV CHAT on HPV vaccine uptake in seven family medicine and pediatric clinics in a large urban health system (USA).

**Methods:**

We used a quasi-experimental design, where the eligible population included people aged 9–26 years with at least one encounter in June 2020–February 2023 at one of the participating community health clinics. We used interrupted time-series analysis to assess changes in the prevalence of HPV vaccine uptake. We used segmented Poisson regression with a log link function to estimate prevalence ratios (PR) and 95% confidence limits (CL) for level (immediate) and slope (over time) changes with adjustment for seasonality using Fourier transformation.

**Results:**

Our study population comprised 60,328 observations in which the median age was 17 years (interquartile range: 13–21). A majority (58%) were female and 87% were racial/ethnic minorities. Overall, we observed no sizeable effect of the intervention on HPV vaccination uptake. Nonetheless, heterogeneity was observed by age group with modest increases in individuals aged 9–12 and 13–17 years.

**Conclusion:**

Our provider feedback intervention had minimal effect on increasing prevalence of HPV vaccination in seven family medicine and pediatric clinics. Novel strategies are needed to address provider barriers related to HPV vaccination.

## 1 Introduction

Human papillomavirus (HPV) vaccination guidelines in the U.S. were first introduced in 2006 for females and 2009 for males, but vaccine coverage remains suboptimal. (Pingali et al., 2023) HPV vaccine initiation was 76%, and completion was 63% in 2022 among adolescents aged 13–17 years. (Pingali et al., 2023) These estimates remain below the Healthy People 2030 national target of 80% completion. (United States Preventive Services Task Force et al., 2018, Office of Disease Prevention and Health Promotion. (n.d.)) Suboptimal uptake is partly attributable to vaccine hesitancy resulting from pervasive misinformation about HPV vaccine effectiveness, safety, and behavioral implications. (National Institute for Health and Care Excellence NICE, 2022) Strong recommendation from a healthcare provider about the effectiveness, safety, and routineness of HPV vaccination can increase acceptability and uptake. (Gilkey et al., 2016, National Institute for Health and Care Excellence NICE, 2022) Nevertheless, provider knowledge about HPV vaccination may not be current, and recommendations are often ambiguous and presented with mixed messages. (Leung et al., 2019, Gilkey and McRee, 2016).

Given the importance of provider recommendation and need for improving communication about HPV vaccination, we developed and implemented a provider-focused communication training intervention. This intervention was based on concepts of motivational interviewing and presumptive recommendation, which assumes parents are ready to vaccinate. Presumptive recommendation may increase vaccine uptake compared with conversational language. (Opel et al., 2013, Brewer et al., 2017) Nevertheless, the effectiveness of this intervention has not been evaluated. Evidence generated from such evaluation may be useful for care delivery organizations that are deliberating strategies for improving HPV vaccine uptake, particularly considering that provider education strategies often improve provider-related process outcomes (e.g., knowledge, self-efficacy, and comfort with recommending vaccination) but have inconsistent effects on HPV vaccination coverage. (Kumar et al., 2019, Leung et al., 2019, Wu et al., 2023) Therefore, we aimed to evaluate the effect of a provider communication intervention titled, “HPV: **C**ommunicating about **H**PV to **A**dults and **T**eens” (HPV CHAT) on HPV vaccine initiation in seven family medicine and pediatric clinics that are part of a large urban health system.

## 2 Methods

### 2.1 Study population

JPS Health Network (JPS) is a large urban safety-net health system in Tarrant County, Texas, USA (>2 million population (United States Census Bureau, 2023)) and primary source of healthcare for socioeconomically marginalized populations in the county. (Institute of Medicine, 2000.) The network comprises a 583-bed main hospital and 40 community health clinics, of which 15 are accredited Patient-Centered Medical Homes. We used the JPS Health Network electronic health record (EHR) system to identify the study population for this quasi-experimental study. (Bernal et al., 2017) The eligible population included individuals aged 9–26 years who completed at least one encounter between June 2020 and February 2023 at one of the seven participating community health clinics within the network (i.e., four family medicine, one pediatric, and two school-based community health clinics).

### 2.2 Intervention

We developed a provider communication intervention, HPV CHAT, which included a 20-minute communication training module delivered as an on-demand video and clinic-level audit and feedback on HPV vaccine uptake. The provider training module covered HPV vaccination guidelines, HPV vaccination as cancer prevention, presumptive recommendation, and motivational interviewing for parents or patients with vaccine hesitancy. HPV CHAT included common parent and patient questions or concerns with examples of evidence-based responses to motivate vaccination. (Malo et al., 2016, Opel et al., 2013, Brewer et al., 2017, Shah et al., 2019) The training video was recorded by a pediatrician (author SM) and offered asynchronously to physicians, advanced practice practitioners, nurses, and medical assistants. Clinic-level audit and feedback comprised monthly data reports (i.e., May, June, July 2022) with HPV vaccination uptake performance compared with the corresponding prior year (i.e., May, June, July 2021) stratified by sex and age group (i.e., 9–12, 13–17, 18–26 years). Reports were electronically delivered to operational and clinic teams by The intervention was initiated in May 2022 with monthly clinic-level audit and feedback through July 2022. We provided clinic-level and individual-level incentives to increase intervention adherence. The clinic-level incentive was a clinic lunch when 50% of the providers within a clinic completed the module. The individual-level incentive was a water bottle (∼$35) for providers who completed the module. This study was reviewed by the North Texas Regional Institutional Review Board and determined to be non-human subject research.

### 2.3 Outcome

Our outcome of interest was prevalence of HPV vaccination among patients aged 9–26 years at the seven participating JPS community health clinics. We defined prevalent vaccination for each patient as documentation of at least one dose of HPV vaccination in EHR including vaccination administered outside the participating clinics or our health system. Specifically, documentation was based on Current Procedural Terminology codes (90649, 90650, 90651) in the EHR or documentation by provider in immunization records or health maintenance records. Patient-level vaccination data were subsequently aggregated to clinic-level data, and prevalence was measured monthly using repeated cross-sections of the eligible population. The denominator of monthly prevalence was the total number of eligible patients aged 9–26 years who completed at least one encounter at any of the participating clinics within the month. The numerator was the number of patients with documentation of at least one dose of HPV vaccine by the end of the month among patients in the denominator.

### 2.4 Data analysis

We used interrupted time-series analysis to assess the effect of intervention (i.e., provider training with clinic-level audit and feedback) on the prevalence of HPV vaccination, where the pre-intervention period was June 2020 to May 2022 and post-intervention period was June 2022 to February 2023. We estimated prevalence ratios (PRs) and 95% confidence limits (CL) for slope and level changes after intervention using segmented regression based on generalized linear models with Poisson distributions and log link function. (Bernal et al., 2017) We adjusted for seasonality for each 12-month period using Fourier transformation and used the scale parameter based on the ratio of Pearson χ (Office of Disease Prevention and Health Promotion. (n.d.)) statistics to the corresponding degrees of freedom to adjust for over dispersion. (Bernal et al., 2017) In addition, we stratified analyses by age group and sex using the same analytic approach to assess potential effect heterogeneity on a multiplicative scale given *a priori* evidence of heterogeneity in HPV vaccine uptake by age and sex. (Pingali et al., 2022, Lu et al., 2021) We quantified heterogeneity using the ratio of prevalence ratios (RPRs) with corresponding 95% CL, (Altman and Bland, 2003) where RPRs indicate the magnitude of heterogeneity when comparing one subgroup with a reference subgroup. All analyses were performed using Stata 17 (StataCorp, College Station, TX).

## 3 Results

### 3.1 Demographics

Our study population comprised 60,328 observations with visits at one of the seven participating community health clinics, where 44,648 observations occurred pre-intervention (June 1, 2020 to May 31, 2022) and 15,680 observations occurred post-intervention (June 1, 2022 to February 28, 2023). Table 1 summarizes distributions of the sociodemographic characteristics of our study population by intervention period. The distribution of sociodemographic characteristics was generally similar except for modestly higher proportion of patients with Medicaid or Medicare coverage in the post-intervention period. Briefly, median age of pre-intervention patients was 17 years (interquartile range = 13–21), 58% were female, 56% were Hispanic, and 60% had Medicaid or Medicare coverage. Median age of post-intervention patients was 16 years (interquartile range = 12–21), 58% were female, 58% were Hispanic, and 69% had Medicaid or Medicare coverage.

**Table 1:** Sociodemographic characteristics of primary care patients aged 9 to 26 years at participating clinics in Tarrant County, Texas between June 2020 and February 2023.

	Pre-intervention^a^ (*n* = 44,648) *n* (%)	Post-intervention^b^ (*n* = 15,680) *n* (%)
Age (years)		
Median (interquartile range)	17 (13–21)	16 (12–21)
9–12	11,625 (26)	4,585 (29)
13–17	13,682 (31)	5,102 (33)
18–26	19,341 (43)	5,993 (38)

**Sex**		
Female	25,946 (58)	9,110 (58)
Male	18,699 (42)	6,568 (42)
Missing	3 (0.01)	2 (0.01)

**Race/Ethnicity**		
Non-Hispanic White	5,787 (13)	1,835 (12)
Non-Hispanic Black	7,649 (17)	2,778 (18)
Hispanic	24,990 (56)	9,128 (58)
Non-Hispanic other	5,597 (13)	1,754 (11)
Missing	625 (1.4)	185 (1.2)

**Insurance**		
Medicaid	26,446 (59)	10,755 (69)
Medicare	327 (0.73)	83 (0.53)
Private insurance	7,517 (17)	2,092 (13)
Uninsured	5,941 (13)	1,399 (8.9)
Hospital-based medical assistance program	4,230 (9.5)	1,347 (8.6)
Other^c^	187 (0.42)	4 (0.03)

a Pre-intervention: 06/01/2020–05/31/2022.

b Post-intervention: 06/01/2022–02/28/2023.

c Veteran, inmate, worker’s compensation insurance.

### 3.2 Effect of the intervention

**Fig. 1:**
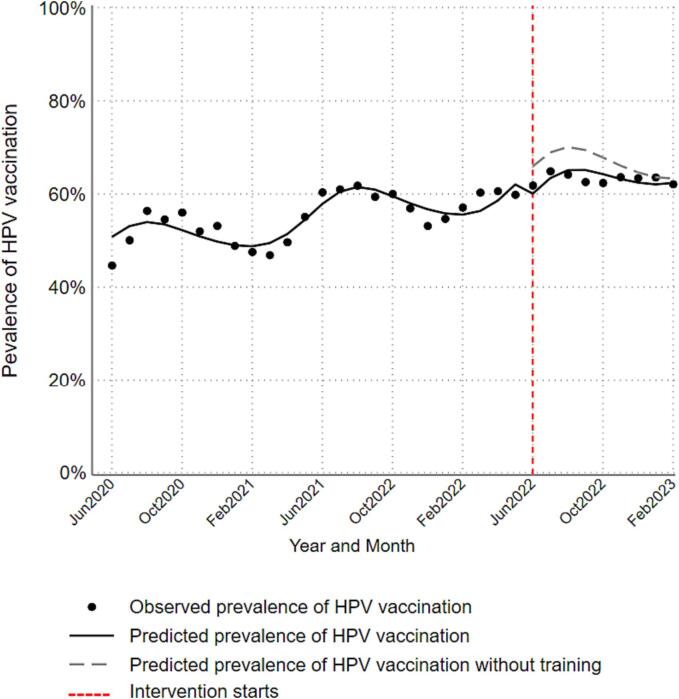
Overall time trends in HPV vaccination prevalence among primary care patients aged 9 to 26 years at participating community health clinics in Tarrant County, Texas between June 2020 and February 2023.

**Fig. 2:**
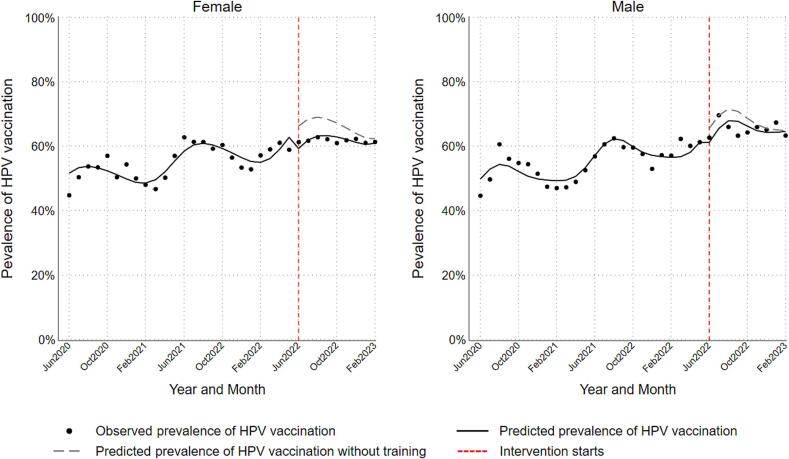
Sex-stratified time trends in HPV vaccination prevalence among primary care patients aged 9 to 26 years at participating community health clinics in Tarrant County, Texas between June 2020 and February 2023.

**Fig. 3:**
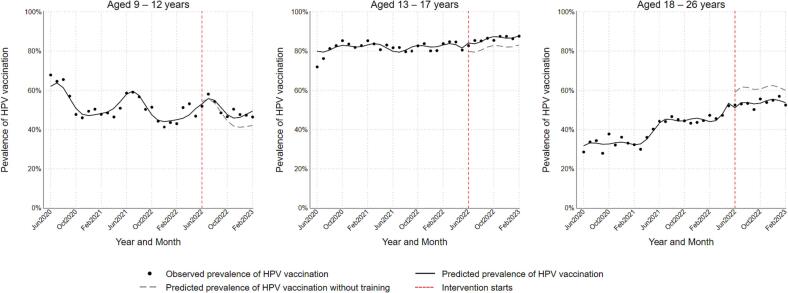
Age-stratified time trends in HPV vaccination prevalence among primary care patients aged 9 to 26 years at participating community health clinics in Tarrant County, Texas between June 2020 and February 2023.

Participation in the intervention was 62% among nurses, 53% among medical assistants, and 38% among physicians and advanced practice providers. Fig. 1 illustrates time trends in monthly overall observed prevalence of HPV vaccination and predicted prevalence of vaccination. Fig. 2 (age-specific) and Fig. 3 (sex-specific) illustrate subgroup-specific time trends in monthly observed and predicted prevalence of HPV vaccination. Table 2 summarizes overall and subgroup-specific level and slope change. Overall, the level change prevalence ratio was 0.90 (95% CL: 0.83, 0.98) and slope change prevalence ratio was 1.01 (95% CL: 1.00, 1.02). Comparisons of level changes suggested that adolescents aged 13–17 years had 1.09 times the prevalence of HPV vaccination after the intervention compared with children aged 9–12 years (RPR = 1.09, 95% CL: 0.95, 1.25). In contrast, adults aged 18–26 years had 0.90 times the prevalence of vaccination after the intervention compared with children aged 9–12 years (RPR = 0.90, 95% CL: 0.76, 1.06). Males had 1.04 times the prevalence of vaccination compared with females (RPR = 1.04, 95% CL: 0.92, 1.19). Comparison of slopes suggested that adolescents aged 13–17 years and adults aged 18–26 years had 0.98 times the increase in prevalence of HPV vaccination over time after the intervention compared with children aged 9–12 years (RPR = 0.98, 95% CL: 0.96, 1.00). The increase in prevalence of vaccination over time after the intervention were comparable between males and females.

**Table 2:** Overall and subgroup-specific effects of provider education intervention on HPV vaccination prevalence among primary care patients aged 9 to 26 years at participating community health clinics in Tarrant County, Texas between June 2020 and February 2023.

	Level change		Slope change	
	PR^a^ (95% CL^b^)	RPR^c^ (95% CL^b^)	PR^a^ (95% CL^b^)	RPR^c^ (95% CL^b^)
Overall	0.90 (0.83, 0.98)		1.01 (1.00, 1.02)	

**Age** (years)				
9–12	0.96 (0.84, 1.09)	1.00 (reference)	1.02 (1.00, 1.04)	1.00 (reference)
13–17	1.05 (1.01, 1.10)	1.10 (0.95, 1.25)	1.00 (0.99, 1.01)	0.98 (0.96, 1.00)
18–26	0.86 (0.78, 0.96)	0.90 (0.76, 1.06)	1.00 (0.99, 1.02)	0.98 (0.96, 1.00)

**Sex**				
Female	0.89 (0.81, 0.97)	1.00 (reference)	1.01 (1.00, 1.03)	1.00 (reference)
Male	0.93 (0.84, 1.02)	1.04 (0.92, 1.19)	1.01 (0.99, 1.03)	1.00 (0.98, 1.03)

a PR: HPV vaccination prevalence ratio comparing post-intervention and pre-intervention periods.

b CL: confidence limits.

c RPR: Ratio of prevalence ratios.

## 4 Discussion

Our overall results suggest a modest decrease in HPV vaccination prevalence immediately after a brief provider communication training with clinic-level audit and feedback at seven community health clinics. This level change was followed by small gradual increase in HPV vaccination prevalence over time after the intervention. We observed potential effect heterogeneity in level change by age group, but the quantification of effect heterogeneity was too imprecise for definitive conclusions. In addition, we did not observe notable heterogeneity in slope change by age or sex.

### 4.1 Limitations

Several limitations should be considered when interpreting our results. Our pre-intervention period covered between June 2020 and May 2022, which includes a period with pandemic-related healthcare disruptions that could function as a co-intervention and induce confounding bias. For example, immunizations declined after the U.S. declared a public health emergency for COVID-19 in March 2020 and catchup efforts began after COVID-19 vaccines were available in December 2020. (Spencer et al., 2022, Pingali et al., 2021) HPV vaccination prevalence during the pre-intervention period could be lower than typical, and thus the intervention could be even less effective than observed. Another source of confounding bias could be temporal drift in population characteristics. Despite generally consistent distributions of sociodemographic characteristics during our study period, the proportion of patients without insurance decreased from 19% pre-intervention to 11% post-intervention among patients aged 18–26 years. Given that individuals without insurance have lower vaccine uptake, (Lu et al., 2015) HPV vaccination prevalence could be lower in the pre-intervention period. Similar to the consequences of confounding bias from pandemic-related disruptions, the intervention could be even less effective than observed given the temporal shift in insurance status.

Outcome misclassification is another potential bias that should be considered. HPV vaccination status was based on electronic health records for our health system, but vaccination outside of our health system may not be documented (i.e., false-negative), and thus the prevalence of HPV vaccination could be underestimated. If specificity of outcome classification was 100% (i.e., no false-positive documentation of HPV vaccination status, which is plausible considering our outcome was based on documented orders in EHR) and sensitivity was <100% but precisely equal before and after the intervention, then misclassification would be inconsequential in our study. (Rothman and Lash, 2020, Howe et al., 2009) Nevertheless, an assumption of precisely equal sensitivity is unrealistic and even minor differences may have notable consequences on our estimates. For example, using standard formulae for outcome misclassification, (Rothman and Lash, 2020, Howe et al., 2009) if we assume perfect specificity of HPV vaccination status before and after the intervention, but sensitivity was 100% before the intervention and 99% after the intervention, then the prevalence ratio may be upward biased by 1%, which would entirely explain our estimates for the intervention slope. Unfortunately, we lack data about HPV vaccination outside our health system and can only speculate about potential consequences.

Lastly, our follow-up duration was limited to a 9-month period after intervention, and thus our findings are more insightful about short-term effects of the intervention. Longer follow-up is needed to evaluate potential long-term effects. Nevertheless, educational interventions are susceptible to fadeout of intervention effects, where longer duration of follow-up reveals a decline. (Bailey et al., 2020) Therefore, longer follow-up may not necessarily reveal intervention effectiveness.

### 4.2 Cumulative evidence

Several studies reported evaluations of diverse provider-focused HPV education interventions using audit and feedback. (Wu et al., 2023) We identified four studies with interventions similar to ours (summarized in Table 3) that focused on different age groups between 9 and 26 years and all studies reported no or minimal effect on HPV vaccination initiation. (Bradley-Ewing et al., 2022, Gilkey et al., 2019, Irving et al., 2018, Perkins et al., 2015) The length of educational training ranged from a single session (30–60 min) to 6–8 sessions over one year (>180 min). Our HPV CHAT intervention was a comparatively brief (20 min) on-demand didactic video. This format and duration were based on guidance from a local advisory board of providers during intervention development. The education components included similar content about HPV infection, HPV vaccines, and communication strategies for HPV recommendation. The audit and feedback strategies varied substantially and included individual provider-level or clinic-level data on missed opportunities at vaccine-eligible preventive care visits, comparison of individual data to other providers in their practice, and goal setting.

**Table 3:** Characteristics of intervention studies in the United States published between 2013 and 2022 with provider education and audit and feedback to increase HPV vaccination among adolescents and young adults.

Authors	Study Years	Setting	Patient Population	Intervention	Baseline Prevalence of Vaccine Initiation
Irving et al. (Irving et al., 2018)	2011–2016	12 family medicine and pediatrics departments within an integrated health system	• Ages 11–17 years with at least 6 months of continuous health plan enrollment • Sociodemographic characteristics not reported	**Education:** 30-minute education session combining information on HPV infection and parental communication strategies. Education included Centers for Disease Control and Prevention-developed “You are the Key to Cancer Prevention” materials. **Clinic-level audit and feedback:** Clinic-level and provider-level data on HPV vaccine coverage rates, compared with rates of MenACWY and Tdap in the same populations, and missed opportunities for HPV vaccination.	**Ages 11–12 years by sex** 40% and 26% among girls and boys, respectively. **Ages 13–17 years by sex** 68% and 22% among girls and boys, respectively
Perkins et al. (Perkins et al., 2015)	2011–2013	Outpatient pediatric clinic of an urban academic medical center and 7 affiliated FQHC centers	• Ages 11–21 years • 70% racial and ethnic minority • 30% English was not their primary language • 75% public insurance	**Education:** 6–8 sessions over one year conducted by an HPV physician-educator. Education covered HPV-related cancers, vaccine efficacy, and safety and use of basic motivational interviewing principles with vaccine-hesitant parents. **Individual-level audit and feedback:** Compared provider and practice HPV vaccination rates to state and national rates. Participants received individual reports showing performance compared to other providers in their practice.	**Ages 11–21 years** 68% of girls and 9.4% of boys
Gilkey et al. (Gilkey et al., 2019)	2017	25 clinics within a pediatric health care system	• Ages 12–14 years • Sociodemographic characteristics not reported	**Education:** 1-hour, in-clinic training session led by a high performing pediatrician (i.e., ≥70% HPV initiation). Session taught epidemiology of HPV, the need to improve HPV vaccine coverage, vaccine safety, and the importance of delivering high-quality recommendations. **Individual-level audit and feedback:** Individual assessment and feedback on HPV vaccination coverage at 3 time points. The report card was used to encourage physicians to set a goal to raise HPV vaccination coverage over the 6-month project period by vaccinating at least 10% of their 12 to 14 year old patients who had not initiated HPV vaccination.	**Ages 12–14 years** 53% in the intervention arm, 45% in the control arm
Bradley-Ewing et al. (Bradley-Ewing et al., 2022)	2018–2019	4 practices with lower than national average HPV vaccination rates	• Ages 9–17 years • 63% White, 27% African American, 10% Other • 68% private insurance, 29% Medicaid/Medicare, 2.5% other/none	**Education:** Provider communication training (e.g., presumptive, bundled with other vaccines and indicating a sense of urgency, cancer prevention, and importance) **Audit and feedback (level not clear):** Assessment and feedback, not clear if individual-level or clinic-level or frequency.	**Ages 9–17 years** Range of 53–61% across 4 practices
Current	2020–2023	4 family medicine 1 pediatric, and 2 school-based community health clinics within an urban safety-net health system	• Ages 9–26 years • 13% non-Hispanic White, 17% non-Hispanic Black, 56% Hispanic, 13% non-Hispanic other 17% private insurance, 59% Medicaid, 13% uninsured, 10% with hospital-based medical assistance program	**Education:** 20-minute communication training module delivered by a local pediatrician and available as an on-demand video. The training covered HPV vaccination guidelines, HPV vaccination as cancer prevention, presumptive recommendation, and motivational interviewing for parents or patients with vaccine hesitancy. **Audit and feedback:** Clinic-level audit and feedback comprised baseline and monthly data reports with HPV vaccination uptake performance stratified by sex and age group. Reports were emailed to operational and clinic teams.	**Ages 9–26 years** 60% **Ages 9–12 years** 47% **Ages 13–17 years** 81% **Ages 18–26 years** 52%

Limited effects of provider-focused HPV education interventions using audit and feedback strategies are consistently reported across studies despite variation in study period, intervention intensity, demographic variation between study populations, and baseline HPV vaccination coverage. Notably, baseline prevalence of HPV vaccination was relatively high in our population (60% overall among patients aged 9–26 years). Our health system had existing strategies to support HPV vaccination before implementation of the current intervention, such as EHR-based health maintenance reminders and navigation efforts by panel care coordinators. These existing efforts, which had no overlap with HPV CHAT, were sustained during the intervention and no other initiatives were implemented during the study period. The remaining unvaccinated patients may have high vaccine hesitancy, possibly exacerbated by backlash against the COVID-19 vaccine during our study period, or parents/patients may have preferred to delay routinely recommended vaccines to prioritize COVID-19 vaccine. (Larson et al., 2022) In addition, participation in our intervention was relatively low (38%) among physicians and advanced practice providers compared with nurses (62%), which could have attenuated the intervention effect. The lack of intervention effect observed in our study and studies with similarly high baseline prevalence of HPV vaccination may also be attributable to a plateau effect, where vaccine uptake reached a level that requires more targeted and innovative strategies to address remaining barriers. In addition, design and analytic errors in prior studies, such as lack of adjustment for confounding bias (Clarke et al., 2019, Spiegelman et al., 2016) and use of change from baseline as the outcome measure, (Fu and Holmer, 2015, Tennant et al., 2022) impede proper interpretation and comparison with our study.

### 4.3 Implications

Despite evidence that provider recommendation is strongly associated with HPV vaccination, results from our study and other studies suggest that existing provider training interventions using audit and feedback strategies have limited effect on HPV vaccination. Efforts to identify potential explanations for the limited effects of audit and feedback across studies, such as low intervention adherence, may be useful for understanding whether this approach can be modified for enhanced effects. If modification is infeasible or unsuccessful, then alternate strategies may need to be explored for increasing HPV vaccination. For example, a pilot study reported that immersive virtual reality training may change behavior to a greater extent compared to traditional educational videos. (Real et al., 2022) In addition, given the continuously increasing workload of healthcare providers, strategies that reduce provider workload may need to be explored. For example, EHR-integrated decision support such as non-interruptive provider reminders or patient education and reminders through patient portals may need to be tested in diverse settings. (Lake et al., 2019, Stephens et al., 2019, Vinci et al., 2022, Molokwu et al., 2023) Innovative strategies are likely needed to increase HPV vaccination initiation and completion to reach the Healthy People 2030 target of 80% coverage and ensure health equity across population subgroups.

Funding Sources: This project was supported by The University of Texas MD Anderson Cancer Center.

## CRediT authorship contribution statement

**Rachel J. Meadows:** Writing – review & editing, Writing – original draft, Project administration, Investigation, Conceptualization. **Aaron W. Gehr:** Writing – review & editing, Writing – original draft, Investigation. **Yan Lu:** Writing – review & editing, Writing – original draft, Visualization, Investigation, Formal analysis. **Grace Maynard:** Writing – review & editing, Project administration, Investigation. **Idara N. Akpan:** Writing – review & editing, Investigation. **Tanjila Taskin:** Writing – review & editing, Investigation. **Kimberly G. Fulda:** Writing – review & editing, Investigation. **Divya Patel:** Writing – review & editing, Investigation. **Sarah Matches:** Writing – review & editing, Resources. **Rohit P. Ojha:** Writing – review & editing, Methodology. **Erika L. Thompson:** Writing – review & editing, Writing – original draft, Supervision, Funding acquisition, Conceptualization.

## Declaration of competing interest

The authors declare the following financial interests/personal relationships which may be considered as potential competing interests: Erika Thompson is funded from the Merck Investigator Initiated Studies Program and serves as a consultant for Merck on HPV vaccination, neither is related to the current study. No potential conflict of interest was reported by the other authors..

## Data Availability

The authors do not have permission to share data.
